# Ethylenediaminetetraacetic Acid Enhances Vancomycin and Reactive Oxygen Species–Mediated Killing of Vancomycin-Intermediate *Staphylococcus aureus*

**DOI:** 10.1093/ofid/ofaf291

**Published:** 2025-05-13

**Authors:** Joshua Olson, Anuj K Khetarpal, Allen Jankeel, Arianna Lorenzana, Victor Nizet, George Sakoulas, Erlinda R Ulloa

**Affiliations:** Department of Pediatrics, University of California, San Diego School of Medicine, La Jolla, California, USA; Department of Pediatrics, University of California, Irvine School of Medicine, Irvine, California, USA; Department of Pediatrics, University of California, Irvine School of Medicine, Irvine, California, USA; Department of Pediatrics, University of California, Irvine School of Medicine, Irvine, California, USA; Department of Pediatrics, University of California, San Diego School of Medicine, La Jolla, California, USA; Department of Pediatrics, Collaborative to Halt Antibiotic-Resistant Microbes, University of California, San Diego School of Medicine, La Jolla, California, USA; Skaggs School of Pharmacy, University of California, San Diego, La Jolla, California, USA; Department of Pediatrics, University of California, San Diego School of Medicine, La Jolla, California, USA; Department of Pediatrics, Collaborative to Halt Antibiotic-Resistant Microbes, University of California, San Diego School of Medicine, La Jolla, California, USA; Division of Infectious Diseases, Sharp Rees Stealy Medical Group, San Diego, California, USA; Department of Pediatrics, University of California, Irvine School of Medicine, Irvine, California, USA; Division of Infectious Diseases, Children's Hospital of Orange County, Orange, California, USA

**Keywords:** antibiotic tolerance, EDTA, ROS, vancomycin, VISA

## Abstract

**Background:**

The emergence of antibiotic-tolerant staphylococcal strains, such as vancomycin-intermediate *Staphylococcus aureus* (VISA), poses a significant healthcare challenge and complicates treatment regimens. VISA often exhibits mutations in Krebs cycle enzymes, promoting anaerobic metabolism under physiological conditions and reducing susceptibility to antibiotics and innate immune defenses—factors not typically captured in standard susceptibility testing. Building on these findings, we investigated ethylenediaminetetraacetic acid (EDTA), a chelator that enhances reactive oxygen species (ROS) production, as an adjunct to vancomycin for combating VISA infections.

**Methods:**

RNA sequencing analysis compared gene expression of a well-characterized VISA strain (D712) under physiological versus nutrient-rich conditions. Hydrogen peroxide (H_2_O_2_) killing assays were conducted with and without the hydroxyl radical quencher thiourea, while neutrophil killing assays used ROS scavenger butylated hydroxyanisole (BHA). A murine bacteremia model assessed the effects of vancomycin, EDTA, or their combination on VISA.

**Results:**

VISA exhibited downregulation of Krebs cycle enzymes and other genes associated with resistance to iron and ROS-mediated killing under physiological conditions. EDTA, alone or with vancomycin, improved H_2_O_2_-mediated killing compared to vancomycin alone, a response counteracted by thiourea. The combination of EDTA and vancomycin enhanced neutrophil killing of VISA more effectively than either treatment alone, an effect negated by BHA. In vivo, EDTA enhanced vancomycin activity against VISA.

**Conclusions:**

EDTA potentiates vancomycin efficacy against VISA in vivo and enhances susceptibility to H_2_O_2_ and neutrophil killing. Reduced expression of Krebs cycle enzymes in VISA suggests that EDTA promotes ROS-mediated bacterial killing, targeting a key mechanism by which persistent staphylococci evade host defenses.

## Teaser

Ethylenediaminetetraacetic acid (EDTA) potentiates vancomycin efficacy against vancomycin-intermediate *Staphylococcus aureus* (VISA) by enhancing susceptibility to reactive oxygen species and neutrophil killing, overcoming VISA's anaerobic metabolism and resistance mechanisms. EDTA may serve as a potential adjunct therapy for antibiotic-tolerant VISA infections.

While vancomycin remains a cornerstone treatment for methicillin-resistant *Staphylococcus aureus* (MRSA) infections, its efficacy as monotherapy is often suboptimal in complicated infections, even though >97% of isolates show in vitro susceptibility [1]. This discrepancy between in vitro results and clinical outcomes underscores the need to account for *Staphylococcus aureus*' remarkable phenotypic plasticity in response to environmental changes, including variations in nutrient availability, oxygen levels, pH, metal ions, and antibiotics [2]. Notably, the adaptive capacity of vancomycin-intermediate *S aureus* (VISA) strains often contributes to treatment failures, as these strains survive vancomycin through physiological and metabolic adaptations not captured by standard susceptibility testing [3].

Antibiotic susceptibility is traditionally assessed using cation-adjusted Mueller-Hinton broth (CA-MHB), a nutrient-rich bacteriological medium supplemented with divalent ions such as calcium (Ca^2+^) and magnesium (Mg^2+^). However, several studies have shown that CA-MHB fails to recapitulate the physiological conditions bacteria encounter in vivo [4–8]. Clinical isolates from vancomycin-refractory staphylococcal infections are often misclassified as vancomycin-susceptible in CA-MHB, but display vancomycin-intermediate phenotypes in physiological media (eg, RPMI medium + 10% Luria-Bertani broth [LB]) [3, 9]. This discrepancy highlights the phenotypic plasticity of *S aureus* and the importance of identifying ways to more accurately model their in vivo behavior. In particular, VISA strains undergo metabolic and transcriptional changes under physiological conditions that enhance vancomycin tolerance [10–12]. Such adaptations include alterations in cell wall thickness, membrane potential, and expression of stress response genes—mechanisms not fully captured in standard CA-MHB testing [10–12].

Iron plays a central role in these metabolic adaptations. While essential for bacterial growth, iron in excess can be harmful, particularly when ferrous iron (Fe^2+^) reacts with hydrogen peroxide (H_2_O_2_) in the Fenton reaction (Fe^2+^ + H_2_O_2_ → Fe^3+^ + OH^—^ + •OH) to produce hydroxyl radicals (•OH) [13], contributing to ROS-mediated killing. Antibiotics can amplify this process by overstimulating the electron transport chain, releasing iron from iron-sulfur clusters, and enhancing Fenton chemistry [14]. Chelators like ethylenediaminetetraacetic acid (EDTA) may potentiate this effect by solubilizing Fe^3+^, thereby increasing the bactericidal potential of ROS [15, 16]. To counteract ROS damage, *S aureus* employs metabolic adaptations, including mutations in Krebs cycle enzymes, which shift the bacteria to an anaerobic metabolic state, reducing generation of hydroxyl radicals (•OH) [10, 11, 17, 18]. This metabolic reprogramming leads to reduced adenosine triphosphate production, which may slow growth and support staphylococcal persistence by limiting antibiotic-induced killing and evading innate immune defenses [10, 11, 19].

These metabolic and therapeutic challenges—including tolerance mechanisms not overcome by standard monotherapies—limit effective VISA treatment. Compounding these barriers is the limited availability of alternative agents like daptomycin and ceftaroline, which may be cost-prohibitive or unavailable in certain regions or healthcare institutions. These challenges underscore the need for new strategies to enhance vancomycin efficacy. In this proof-of-concept study, we evaluate the potential of EDTA as an adjunctive agent, hypothesizing that EDTA enhances VISA susceptibility to ROS-mediated killing under physiological conditions. While previous research has examined chelators like EDTA for disrupting biofilms and improving antibiotic efficacy against resistant strains [15, 16, 20], its specific role in modulating bacterial susceptibility to ROS remains underexplored. By evaluating the effects of EDTA on VISA strains in physiological conditions, this study aims to elucidate how this chelating agent interacts with the phenotypic and metabolic adaptations contributing to vancomycin tolerance, providing insights into its potential as a therapeutic adjunct in clinically relevant settings.

## MATERIALS AND METHODS

### Informed Consent and Institutional Approval

Under a protocol approved by the University of California, San Diego (UCSD) and the University of California, Irvine (UCI) Human Subjects Institutional Review Board, informed consent was obtained for neutrophil studies. Animal experiments were approved by the UCSD and UCI Institutional Animal Care and Use Committee.

### Antibiotics and EDTA

Vancomycin was purchased from the Sharp Memorial Hospital Pharmacy (San Diego, California) and supplied as vials available for clinical use and administration to patients. Stock solutions were prepared in molecular-grade water (Corning Cellgro) and stored at −20°C. For in vivo studies, vancomycin was reconstituted in phosphate-buffered saline (PBS). EDTA was purchased from Promega.

### Bacterial Strains and Antibiotic Susceptibility Assays

Experiments were conducted using well-characterized clinical isolates obtained from patients with persistent VISA bacteremia, including VISA strain D712 [21–23], A5940 [24–27], HIP5836 [24–27], and PC3 [24–28]. Bacteria were grown overnight in Todd-Hewitt broth (THB; Hardy Diagnostics) and stored with 40% glycerol at −80°C. Fresh colonies were streaked onto Todd-Hewitt agar (THA; Hardy Diagnostics) plates each week for all experiments. Broth microdilution antimicrobial susceptibility testing to vancomycin and EDTA were conducted under both standard (10^5^ colony-forming units [CFU]/mL) and high-inoculum (10^7^ CFU/mL) conditions using Mueller-Hinton broth (Difco) bacteriological media based on Clinical and Laboratory Standards Institute guidelines [29].

### Neutrophil Killing Assays

Human neutrophils were freshly isolated from heparinized blood, using Polymorphprep (Axis-Shield) per the manufacturer's instructions, and erythrocytes were lysed with sterile water as previously described [5, 6]. VISA D712 was grown to log phase (∼2 hours) in media alone (untreated), EDTA 150 µg/mL, or vancomycin 1 µg/mL ± EDTA 150 µg/mL. At log phase, bacteria were centrifuged and resuspended in RPMI with 20% pooled human serum for opsonization in RPMI for 10 minutes. Neutrophils were preincubated with and without 10 µM of the reactive oxygen species (ROS) scavenger butylated hydroxyanisole (BHA) for 30 minutes with rotation at 37°C. Neutrophils were infected at a multiplicity of infection of 10 with VISA D712. Plates were centrifuged at 300*g* for 5 minutes to improve contact with bacteria, and subsequently incubated at 37°C in 5% carbon dioxide (CO_2_). Aliquots were collected at 45 minutes and cells were lysed (to account for intracellular bacteria) with ice-cold molecular-grade water supplemented with 0.3% saponin (1:5) on ice for 5 minutes prior to serially diluting in PBS for CFU enumeration on THA plates. The percentage of bacterial survival was determined by comparing the number of surviving bacteria to the input inoculum. These data were collected from at least 3 biological replicates performed in at least technical triplicate using blood from at least 3 individual donors.

### Hydrogen Peroxide Killing Assays

To quantify the impact of EDTA and/or vancomycin preexposures on subsequent resistance to oxidative stress, VISA was grown overnight in tryptic soy broth (TSB) at 37°C with shaking to stationary-growth phase (12–16 hours). Bacteria were then subcultured (1:50) in fresh media with or without sublethal EDTA 100–150 µg/mL or vancomycin 1–2 µg/mL ± EDTA 100–150 µg/mL to an optical density at 600 nm (OD_600_) of 0.40. For the hydroxyl radical quenching experiments, 150 mM thiourea was added to the culture simultaneously with the drugs. After reaching OD_600_ of 0.40, cells were pelleted, washed, and resuspended in PBS. Cultures were diluted to a bacterial inoculum of 5 × 10^5^ CFU/mL and 1.15 mM (w/v) H_2_O_2_ was added, with or without thiourea, prior to incubating at 37°C with shaking. The pH was measured at the start and end of experiments using pH strips. After 45 minutes, aliquots were serially diluted for CFU enumeration on THA plates to determine the mean percent of surviving bacteria (± standard deviation) normalized to H_2_O_2_ untreated conditions.

### Cytochrome C Assays

To assess the impact of EDTA preexposure on the bacterial cell envelope surface charge, bacteria were grown overnight in TSB (20 mL) at 37°C with and without EDTA 150 μg/mL or vancomycin 1 μg/mL, alone or in combination, and washed twice with 20 mM MOPS buffer (4-morpholinepropanesulfonic acid, pH 7). The cells were resuspended in the same buffer to a final OD_600_ of 0.70 in 10 mL, centrifuged (at 3200*g* for 10 minutes), and resuspended in 1 mL of buffer. In a 96-well V-bottom plate (Costar), 190 μL of bacterial culture was mixed with cytochrome c at a final concentration of 0.50 mg/mL. The plate was incubated at room temperature for 10 minutes and centrifuged at 3200*g* for 10 minutes. Supernatant (100 μL) was transferred to a flat-bottom 96-well plate (Costar) and the absorbance measured at 530 nm with the SpectraMax 250 (Molecular Devices). The lower the absorbance, the more negatively charged the VISA D712 cell envelope.

### Murine Bacteremia Model

Cultures of VISA D712 were grown overnight in THB at 37°C with shaking. Overnight cultures were diluted 1:100 in fresh THB and grown to an OD_600_ of 0.40. Bacteria were washed and resuspended in PBS, and 1 × 10^7^ CFU/mL was injected intravenously via tail vein into outbred female CD1 mice (8–10 weeks old, Charles River Laboratories). Mice were then randomly divided into treatment and control groups. Two hours after infection, a single subcutaneous dose of vancomycin (100 mg/kg/day), EDTA (200 mg/kg/day), or vancomycin (100 mg/kg/day) + EDTA (200 mg/kg/day) was administered. Mice were euthanized with CO_2_ 24 hours after infection, followed by cervical dislocation. Kidneys were then harvested for CFU enumeration. Kidneys were weighed and placed in a 2-mL sterile microtube (Sarstedt) containing 1 mL of PBS and 1-mm-diameter silica beads (Biospec). They were subsequently homogenized by shaking twice at 6000 rpm for 60 seconds, using a MagNA Lyser (Roche). Specimens were placed on ice as soon as they were harvested. Aliquots from each tube were serially diluted in PBS for CFU enumeration on THA plates.

### Gene Expression

Publicly available RNA sequencing (RNA-seq) data for VISA strain D712 obtained after 3 hours of growth in bacteriological (CA-MHB) or physiological (RPMI + 10% LB) media were acquired from a previously published study [23]. RNA-seq reads were quality filtered and trimmed using Trim Galore with a minimum phred score of 30. Quality reports were generated with the FastQC function. Trimmed reads were aligned to the VISA D712 genome using HISAT2. Uniquely mapped reads were counted using the summarizeOverlaps function. Data obtained under physiological conditions were normalized, and the differentially expressed genes (DEGs) were identified relative to CA-MHB using the EdgeR package. Only DEGs with at least a 2-fold change in expression and a multiple-hypothesis Benjamini-Hochberg false discovery rate–corrected *P* value <.05 were included.

### Genomic Mutation

The genome of hospital-acquired USA100 lineage VISA strain D712 was previously submitted to the National Center for Biotechnology Information (accession number VFJD00000000.1). Mutations in VISA D712 were identified with breseq (version 0.37.0) and bowtie2 (version 2.4.1) [30, 31] and compared to the complete genome (chromosome and plasmid) of a wild-type USA100 MRSA strain as a reference [32]. Only high-quality mutation calls, as determined by the default parameter settings in breseq (>20× coverage and >90% variant read frequency), were included. To predict protein stability changes of mutations, we used I-Mutant3 and DDGun for predicting the variation of unfolding free energy change upon mutation (ΔΔG) and protein sequence or protein structure when available. Negative ΔΔG corresponds to a predicted decrease to the protein stability [33, 34].

### Statistical Analyses

Statistical analyses were performed using GraphPad Prism software, version 10.2.3. *P* values <.05 were considered statistically significant.

## RESULTS

Given the narrow focus of our investigation, primary studies were conducted using a well-characterized VISA strain, D712 [21–23]. Complementary experiments were performed on 3 additional well-characterized isolates, all from patients with persistent bacteremia [24–28]. In vitro antibiotic activities were assessed at both standard (5 × 10^5^ CFU/mL) and high (2 × 10^7^ CFU/mL) bacterial inocula (Table 1), reflecting conditions relevant to infections like endocarditis [35].

**Table 1.** Minimum Inhibitory Concentration of Vancomycin and Ethylenediaminetetraacetic Acid Against 4 Clinical Isolates of Vancomycin-Intermediate *Staphylococcus aureus*, Tested Under Standard (10^5^ Colony-Forming Units [CFU]/mL) and High (10^7^ CFU/mL) Inoculum Conditions in Mueller-Hinton Broth

Isolate	MIC, mg/L			
	Vancomycin		EDTA	
	10^5^ CFU/mL	10^7^ CFU/mL	10^5^ CFU/mL	10^7^ CFU/mL
D712	2–4	4	128	128
A5940	2–4	4	128	128
5836	4	8	64	128
PC-3	8	16	128	128

Abbreviations: EDTA, ethylenediaminetetraacetic acid; MIC, minimum inhibitory concentration.

### VISA D712 Exhibits Genetic and Transcriptional Adaptations Conferring Resistance to Iron and ROS Killing Under Physiological Conditions

Whole genome sequencing of VISA D712, compared to the wild-type USA 100 MRSA strain, revealed nonsynonymous mutations in key enzymes that were predicted to slow bacterial growth and reduce susceptibility to ROS-mediated killing (Supplementary Table 1). RNA-seq further demonstrated that VISA D712 grown in physiological tissue culture media (RPMI + 10% LB), which more closely mimics human serum and tissue conditions, exhibited significant changes in gene expression compared to growth in standard bacteriological media (CA-MHB) [4–8].

**Figure 1.**
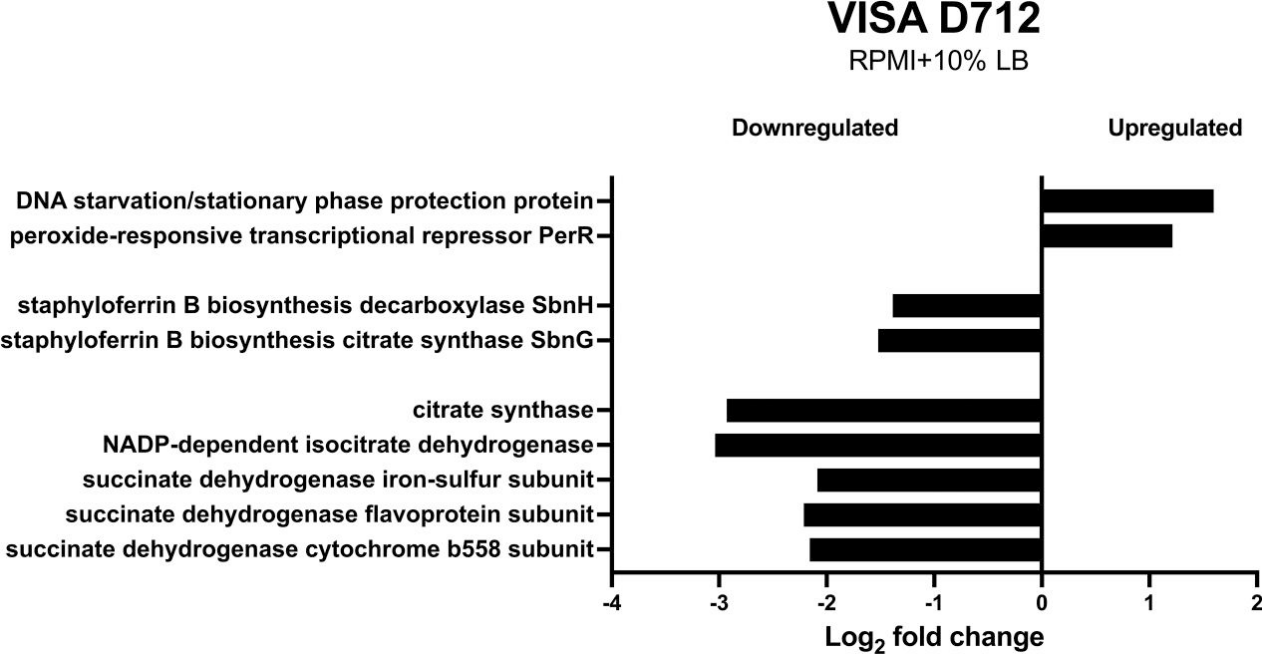
Physiological conditions induce gene expression changes in vancomycin-intermediate *Staphylococcus aureus* (VISA) D712. RNA sequencing analysis was conducted on VISA strain D712 after 3 hours of growth in either standard bacteriological (cation-adjusted Mueller-Hinton broth) or physiological (Roswell Park Memorial Institute medium supplemented with 10% Luria-Bertani broth [RPMI + 10% LB]) conditions. Under physiological conditions, VISA D712 showed a downregulation of genes involved in electron transport, including key Krebs cycle enzymes such as citrate synthase, isocitrate dehydrogenase, and succinate dehydrogenase, which may inhibit energy-dependent processes. Additionally, siderophore genes (eg, staphyloferrin B), responsible for iron uptake, were downregulated, while genes involved in protection against iron-mediated oxidative stress were upregulated. These included PerR, a regulator of intracellular iron and hydrogen peroxide levels, and DNA starvation protection proteins that help prevent hydroxyl radical production via the Fenton reaction.

Key findings included the downregulation of metabolic enzymes, such as citrate synthase, a critical gatekeeper of the Krebs cycle, which showed a 3-fold reduction (Figure 1). Isocitrate dehydrogenase and succinate dehydrogenase were also downregulated by 2–3 logs. This pattern suggests suppression of the tricarboxylic acid cycle, a metabolic state that has been increasingly linked to antibiotic tolerance in *S aureus* [10, 36, 37]. Similarly, the siderophore staphyloferrin B, involved in iron transport, was also downregulated. In contrast, genes involved in protecting against Fenton reaction–induced damage were upregulated. These included the peroxide regulator, which senses H_2_O_2_ and helps manage adaptation to oxidative stress and iron homeostasis [38], as well as DNA starvation protection proteins that sequester Fe^2+^ to protect bacterial DNA from oxidative damage (Figure 1) [39]. For a full list of DEGs, please see the Supplementary material.

### Impact of EDTA and Vancomycin on Oxidative Stress Susceptibility

**Figure 2.**
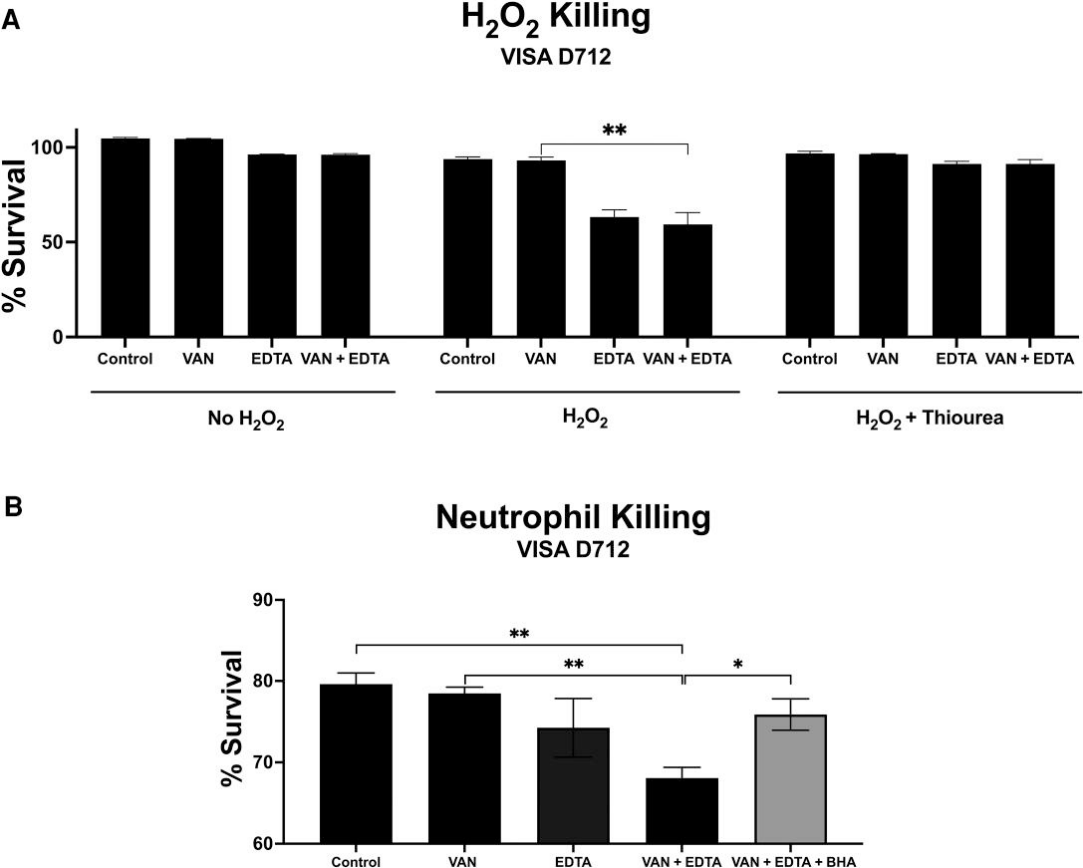
Effect of adjunctive ethylenediaminetetraacetic acid (EDTA) on hydrogen peroxide (H_2_O_2_) and human neutrophil killing of vancomycin-intermediate *Staphylococcus aureus* (VISA) D712. *A*, Adjunctive EDTA (150 μg/mL) or monotherapy increases VISA D712's susceptibility to oxidative stress (H_2_O_2_ killing) compared to vancomycin (VAN, 1 μg/mL) alone at 45 minutes. This effect was reduced by the hydroxyl radical quenching agent thiourea (150 mM), leading to bacterial growth similar to that of bacteria grown without H_2_O_2_ (n = 3). *B*, Adjunctive EDTA increases VISA D712's sensitivity to neutrophil killing (at 45 minutes) in the presence of VAN (1 μg/mL), compared to VAN alone (n = 4). This effect was diminished by the reactive oxygen species scavenger butylated hydroxyanisole (BHA, 10 µM). *P < .05 and **P ≤ .01, 2-tailed Mann-Whitney test.

Building on these insights, we investigated whether EDTA influences oxidative stress susceptibility in VISA D712. Subtherapeutic concentrations of EDTA and vancomycin were used to mimic conditions where neither agent alone achieves effective bacterial killing, allowing us to assess their synergistic effects. H_2_O_2_ killing assays revealed that EDTA alone or combined with vancomycin significantly increased susceptibility to H_2_O_2_-mediated killing compared to vancomycin alone (Figure 2*A*), with this effect abrogated by thiourea, a hydroxyl radical quencher. Similar results were observed in 3 additional VISA isolates (Supplementary Figure 1). All experiments were conducted at physiologic pH.

Neutrophil killing assays demonstrated that the EDTA–vancomycin combination enhanced neutrophil-mediated killing of VISA D712 relative to controls or vancomycin monotherapy (Figure 2*B*). This effect was diminished by the ROS scavenger BHA, further implicating ROS in the observed synergy. Although not statistically significant, EDTA monotherapy also showed a numerical reduction in bacterial counts, suggesting potential standalone activity.

Taken together, these findings suggest that EDTA potentiates the activity of vancomycin against VISA D712 by increasing its susceptibility to ROS-mediated killing, whether from exogenous sources (H_2_O_2_) or that produced by host immune cells (neutrophils).

### In Vivo Efficacy of Vancomycin and EDTA Against VISA D712

**Figure 3.**
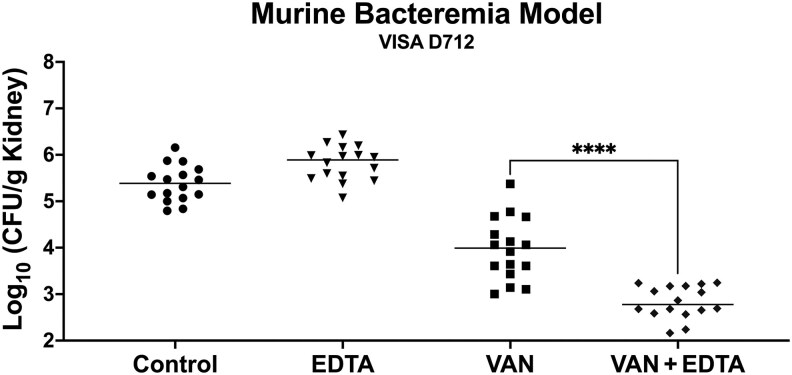
Efficacy of adjunctive ethylenediaminetetraacetic acid (EDTA) with vancomycin (VAN) against vancomycin-intermediate *Staphylococcus aureus* (VISA D712) in a murine bacteremia model. Bacterial counts from kidneys (colony-forming units [CFU]/g) after 24 hours of treatment with VAN (100 mg/kg/day) alone, EDTA (200 mg/kg/day) alone, VAN + EDTA combination, or no treatment (control) are shown. The VAN + EDTA combination significantly reduced recoverable VISA D712 from kidneys compared to VAN alone, EDTA alone, and the control (n = 16). Horizontal bars represent the mean. ****P ≤ .0001, 2-tailed Student *t* test.

To evaluate the therapeutic potential of EDTA in vivo, we tested its efficacy with vancomycin in a murine bacteremia model by quantifying kidney tissue bacterial counts at 24 hours (Figure 3). EDTA exhibited no activity compared to controls, while vancomycin monotherapy provided moderate bacterial reduction. Importantly, the combination of EDTA and vancomycin significantly enhanced bacterial clearance, resulting in an approximately 1.2 log_10_ reduction in recoverable CFU counts.

Although EDTA's potentiation of vancomycin activity in vivo suggests an interaction with innate immune mechanisms, surface charge measurements using cytochrome c binding showed no change in VISA D712 surface charge (Supplementary Figure 2). This suggests that EDTA's effects are likely mediated through ROS enhancement rather than alteration of bacterial surface properties.

## DISCUSSION

The emergence of VISA strains presents a significant clinical challenge, contributing to prolonged bacteremia, increased mortality, and treatment failures, particularly in patients with deep-seated infections. Our study demonstrates that combining EDTA with vancomycin significantly enhances antibacterial therapy against VISA D712, offering a potential strategy to overcome limitations of current treatments. The enhancement appears to stem from increased susceptibility to oxidative stress, driven by ROS produced by immune cells and/or generated as byproducts of antibiotic activity [14]. RNA-seq analysis further revealed downregulation of key Krebs cycle enzymes in VISA D712 under physiological conditions, reflecting a metabolic shift that bolsters resistance to ROS-mediated killing. These findings are consistent with prior research showing that MRSA isolates from persistent bacteremia often reprogram their metabolism to evade oxidative damage [10, 11, 19]. Disruption of central metabolic pathways has also been proposed as a persistence mechanism that promotes survival under antibiotic pressure [10, 36, 37]. These insights underscore the importance of accounting for bacterial metabolic adaptations when designing anti-staphylococcal treatments.

**Figure 4.**
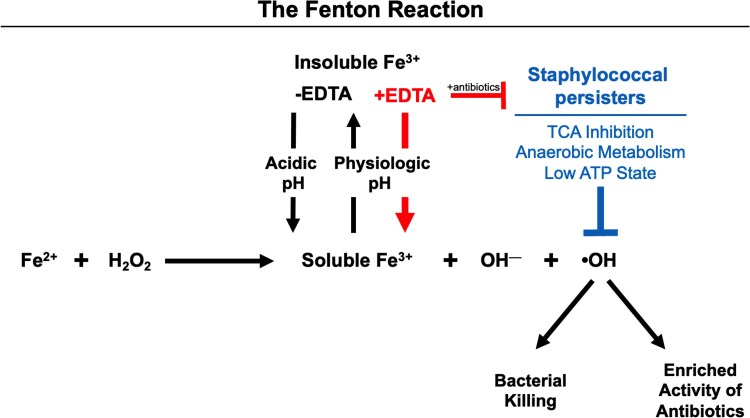
The Fenton reaction, ethylenediaminetetraacetic acid (EDTA), and bacterial adaptation to oxidative stress: a proposed mechanism for enhancing antibiotic efficacy. The Fenton reaction involves the oxidation of ferrous iron (Fe^2+^) by hydrogen peroxide (H_2_O_2_), producing reactive oxygen species (ROS), such as hydroxyl radicals (•OH). This reaction is pH-dependent, with optimal rates at acidic pH (around pH 3). At physiological pH, ferric iron (Fe^3+^) becomes less soluble, potentially slowing the reaction. EDTA may enhance ROS production by increasing the solubility of Fe^3+^, as it has a higher binding affinity for Fe^3+^ compared to Fe^2+^, thus facilitating the Fenton reaction over a broader range of physiological pH conditions. Staphylococcal persisters, which exhibit low adenosine triphosphate (ATP) levels and anaerobic metabolism, often harbor mutations in tricarboxylic acid (TCA) or Krebs cycle enzymes [10, 11]. These enzymes play a key role in generating Fenton-mediated radicals. Overstimulation of the bacterial electron transport chain can release iron from iron-sulfur clusters, potentially activating the Fenton reaction and causing damage to bacterial DNA, proteins, and lipids. EDTA may catalyze this process in the presence of immune factors that generate ROS and antibiotics like vancomycin, potentially overcoming bacterial adaptations to oxidative stress and improving antibiotic efficacy against antibiotic-tolerant and/or -resistant strains.

We propose that EDTA augments vancomycin efficacy by countering VISA's metabolic shift toward an anaerobic state. By solubilizing Fe^3+^, EDTA promotes the Fenton reaction, generating ROS (•OH) that directly kills bacteria and amplifies vancomycin's antibacterial activity (Figure 4). In vivo, this process likely intensifies under physiological conditions, (pH = 7.4), contributing to enhanced bacterial clearance, as observed in our murine bacteremia model.

The benefit of EDTA may be particularly pronounced in persistent infections where metabolic adaptations in MRSA necessitate augmentation of ROS-mediated killing. Preliminary findings suggest that preferential anaerobic growth in *S aureus* bloodstream infections may correlate with a higher risk of treatment failure (author G. S., unpublished observations), indicating a potential clinical metric for considering EDTA-based therapy. Importantly, this strategy may be especially valuable in settings where access to alternative agents like daptomycin or ceftaroline is limited. Vancomycin and EDTA are relatively inexpensive, widely available, and do not require the same infrastructure or cost as newer agents—factors that could expand the therapeutic options for managing VISA infections in resource-constrained healthcare environments.

As a proof-of-concept investigation, this study has several limitations. The small number of isolates tested limits the generalizability of our findings, necessitating further research with a larger and more diverse set of VISA and antibiotic-tolerant staphylococcal strains. Although EDTA is approved by the United States Food and Drug Administration for treating lead poisoning and used off-label for cardiovascular disease [40–42], its use in bacterial infections remains underexplored, requiring careful consideration of its safety and efficacy in this context. While our focus was on ROS-mediated mechanisms, other factors may contribute to the observed synergy between EDTA and vancomycin. These could include biofilm disruption, modulation of host immune responses, and changes in bacterial membrane permeability (despite cytochrome c binding assays showing no direct effect on surface charge). Exploring these possibilities could provide a more comprehensive understanding of EDTA’s role in enhancing vancomycin activity.

We hope this proof-of-concept study will inspire future research to elucidate the mechanisms by which EDTA potentiates vancomycin activity, explore its effects on other antibiotics, and advance clinical studies in serious MRSA bloodstream infections, where vancomycin alone often falls short. Bridging the gap between vancomycin's robust in vitro efficacy and its suboptimal clinical outcomes in MRSA infections could pave the way for improved treatment strategies against antibiotic-tolerant strains like VISA.

## Supplementary Material

ofaf291_Supplementary_Data

## Supplementary Data

Supplementary materials are available at *Open Forum Infectious Diseases* online. Consisting of data provided by the authors to benefit the reader, the posted materials are not copyedited and are the sole responsibility of the authors, so questions or comments should be addressed to the corresponding author.
