# Supplementary material for: Ethylenediaminetetraacetic Acid Enhances Vancomycin and Reactive Oxygen Species–Mediated Killing of Vancomycin-Intermediate Staphylococcus aureus
Source: Open Forum Infect Dis. 2025 May 13;12(6):ofaf291. doi: 10.1093/ofid/ofaf291 (PMC12138334; doi:10.1093/ofid/ofaf291)
Supplement: ofaf291_Supplementary_Data [file ofaf291_supplementary_data.zip › Olson_et_al_Supplemental_tables&figures.docx]

| **Supplemental Table 1.** Genome analysis of VISA D712 identifies non-synonymous mutations in metabolic pathways linked to slower bacterial growth and reduced oxidative stress. | | | | | | |
| --- | --- | --- | --- | --- | --- | --- |
| **Mutation** | **Annotation** | **Gene** | **Description** | **Predicted protein stability** | **I-Mutant3 𝚫𝚫G (Kcal/mol)** | **DDGun 𝚫𝚫G (Kcal/mol)** |
| G→A | V81I (GTT→ATT) | *lpdA* → | dihydrolipoyl dehydrogenase | decrease | -0.46 | -0.2 |
| T→A | F135L (TTT→TTA) | *codY* → | GTP‑sensing pleiotropic transcriptional regulator CodY | decrease | -1.65 | -1.9 |
| G→C | G62A (GGT→GCT) | *sbnA* → | 2,3‑diaminopropionate biosynthesis protein SbnA | decrease | -0.51 | -0.6 |
| A→G | I9V (ATT→GTT) | *gpmI* → | 2,3‑bisphosphoglycerate‑independent phosphoglycerate mutase | decrease | -0.79 | -0.3 |
| G→A | A177T (GCC→ACC) | *argF* → | ornithine carbamoyltransferase | decrease | -0.82 | -0.3 |
| A→T | I137N (ATT→AAT) | *rocD* ← | ornithine‑‑oxo‑acid transaminase | decrease | -2.04 | -0.3 |
| G→A | A316T (GCT→ACT) | *DKK89_RS00470* → | mannitol‑1‑phosphate 5‑dehydrogenase | decrease | -0.59 | -0.3 |
| A→T | N150K (AAT→AAA) | *DKK89_RS02520* ← | NAD(P)H‑dependent oxidoreductase | decrease | -0.6 | -0.4 |
| A→G | R267Q (CGA→CAA) | *DKK89_RS03635* ← | NAD(P)H‑hydrate dehydratase | decrease | -1.06 | -0.1 |


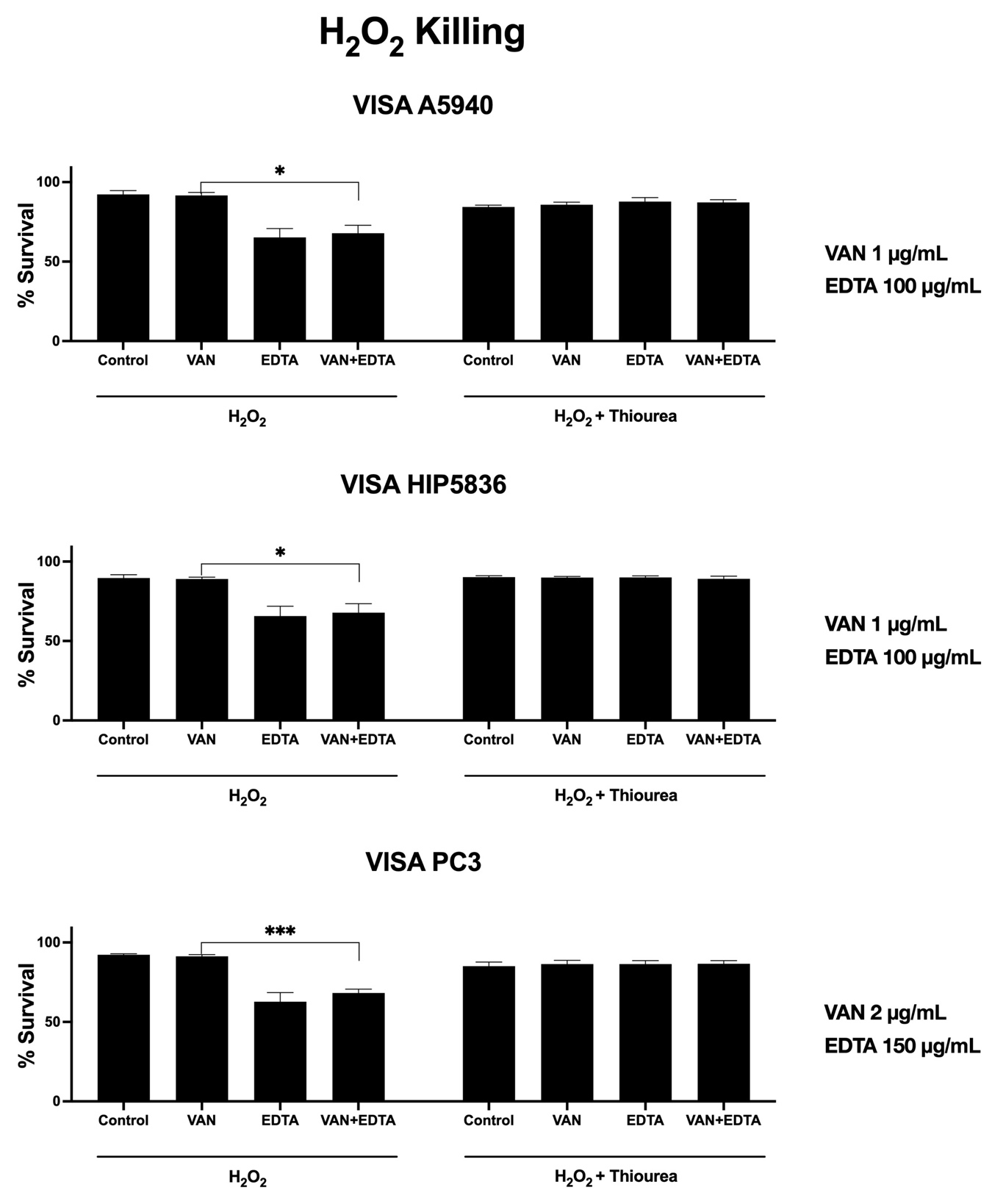


**Supplemental Figure 1. Effect of EDTA on H₂O₂-mediated killing of VISA strains**. Three clinical VISA isolates were exposed to H₂O₂ for 45 minutes under different conditions: control, vancomycin (VAN), EDTA, or their combination (n=3). EDTA, either alone or in combination with VAN, increased susceptibility to H₂O₂-mediated killing compared to VAN alone. The hydroxyl radical scavenger thiourea (150 mM) reduced this effect. *P < 0.05, ***P < 0.001, two-tailed Mann-Whitney test.

**
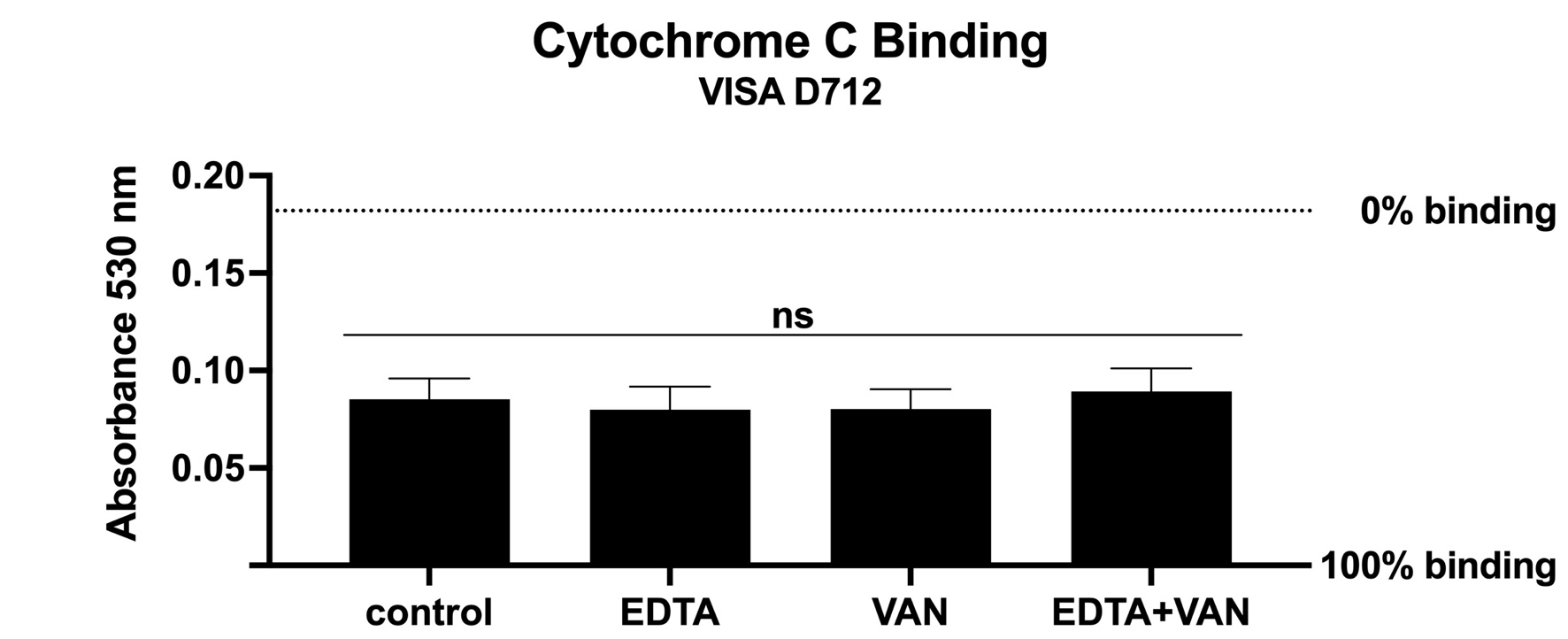
**

**Supplemental Figure 2. Effect of EDTA on surface charge of VISA D712**. Cytochrome c binding assays were performed on VISA strain D712 under various conditions: control, vancomycin (VAN, 1 μg/mL), EDTA (150 μg/mL), or their combination (n=3). Lower absorbance indicates increased cytochrome c binding, reflecting a more negative bacterial surface charge. No significant difference was observed between any of the conditions (NS, not significant; two-tailed Mann-Whitney test).
